# Community Case Study: The Role of Live-Interactive Learning Network in Addressing Community Wide Information Needs Through Show-Me COVID-19 ECHO

**DOI:** 10.3389/fpubh.2022.913747

**Published:** 2022-07-06

**Authors:** Mirna Becevic, Melissa Warne-Griggs, Emmanuelle Wallach, Karen Edison, Rachel Mutrux, Jane A. McElroy, Albert Hsu, Chi-Ren Shyu, Robin Trotman, Kimberly Hoffman

**Affiliations:** ^1^Department of Dermatology, University of Missouri, Columbia, MO, United States; ^2^Missouri Telehealth Network, University of Missouri, Columbia, MO, United States; ^3^Institute for Data Science and Informatics, Columbia, MO, United States; ^4^Family and Community Medicine, University of Missouri, Columbia, MO, United States; ^5^Department of Obstetrics, Gynecology, and Women's Health, University of Missouri, Columbia, MO, United States; ^6^Department of Electrical Engineering and Computer Science, University of Missouri, Columbia, MO, United States; ^7^Infectious Disease, CoxHealth Center, Springfield, MO, United States

**Keywords:** telehealth, project ECHO, virtual learning networks, continuing medical education, COVID-19

## Abstract

After the COVID-19 pandemic reached Missouri, the Show-Me ECHO (Extension for Community Healthcare Outcomes) project initiated COVID-19 ECHO virtual knowledge-sharing networking sessions. These live-interactive weekly sessions inform participants about up-to-date evidence-based recommendations and guidelines through expert didactic lectures followed by real-life case discussions. We conducted a qualitative analysis of pre-session surveys and questions asked during sessions to learn about information needs of community members during first months of public health emergency. This was a pilot project using qualitative analysis of registration questions regarding anticipated COVID-19 community information needs, and participants' questions asked during sessions collected from March 23 until May 4, 2020. We also analyzed participants' satisfaction surveys collected in December 2020. A total of 761 unique participants attended COVID-19 ECHO during the study period. Survey was completed by 692 respondents. Participants asked 315 questions resulting in 797 identified community information needs. Five thematic categories were recognized: patient care, information seeking, minimizing exposure, financial themes, and general comments. Most attendees rated content quality, logistics, and technical operations as good or excellent on a five-point Likert scale. The COVID-19 ECHO model was responsive to the needs of participants by sharing and discussing up-to-date recommendations and guidelines regarding COVID-19. Sessions were well-attended, and the didactic presenters were invited to deliver same or similar presentations at Boone County Medical Society (BCMS) weekly seminars, suggesting the value of the project to healthcare providers and other community members caring for or working with the most vulnerable populations.

## Introduction

To date, the novel viral pathogen SARS-CoV2 has caused over 590 million confirmed cases worldwide, and over 6 million deaths due to COVID-19 (1). Community transmission in the United States (US) was first detected in February 2020, and within just 1 month all 50 states and four US territories reported first cases of COVID-19 (2). By April 7, 2020 it was reported that the US nationwide case doubling time was about 6.5 days (2). The prevalence of COVID-19 rapidly spread to rural areas, increasing from 3.6 per 100,000 people to 43.6 per 100,000 within just 3 weeks in April 2020 (3).

Since the beginning of this pandemic, the public has been deluged with COVID-19 information, misinformation, and disinformation (4). An overwhelming amount of misinformation has hampered compliance with disease prevention and COVID-19 vaccinations, especially in many rural areas of the US (5–7). The explosive spread of this new disease, the rapidly evolving treatment protocols, the lack of a coordinated communication system as well as considerable contradictory reporting all generated a strong need by the medical community for reliable information. Before the COVID-19 pandemic, Americans only received evidence-based care 55% of the time due to exponential growth of medical knowledge that was making it difficult for practitioners to access and implement in practice (8, 9). The pandemic only worsened this gap, and the lack of options and resources overburdened community clinicians who voiced the need for rapid dissemination of evidence-based practices and guidelines, and information on local resources (10).

Swiftly adapting to the pandemic, healthcare providers and administrators led by the University of Missouri's (MU) Missouri Telehealth Network (MTN), recognized the importance of transferring specialized medical knowledge and up-to-date information about COVID-19, especially to rural, isolated, and underserved communities. A model that has been used in many other specialties for live-interactive mentoring of primary care providers (PCPs), Extension for Community Healthcare Outcomes (ECHO), was initiated in the early days of the pandemic, and continues to provide knowledge about COVID-19 to participants (10–12).

The objective of this project was to categorize and analyze participants pre-survey questions as well as questions asked during ECHO sessions to help us better understand information needs of community members during the first months of public health emergency.

### Program Description: COVID-19 ECHO

Project ECHO was created in 2007 by Dr. Sanjeev Arora at the University of New Mexico in order to democratize medical knowledge and share evidence-based recommendations and resources with clinicians “on the front lines of patient care” (8). Project ECHO uses videoconferencing and telemedicine to connect “hub” teams of subject matter experts to participating healthcare providers. The hub team serves as a conduit for accurate and up to date information, often acting as “myth busters.”

On March 23, 2020 the MTN's Show-Me ECHO launched COVID-19 ECHO in Missouri using Zoom video platform (13). The COVID-19 ECHO model was adjusted to provide mentoring and guidance not only to rural healthcare providers, but also to any community member caring for or working with populations at risk. Senior medical director of MTN Show-Me ECHO worked closely with the Missouri Department of Health and Senior Services (DHSS) to recruit hub team members with appropriate expertise. The following criteria are the foundation to successful hub teams: interdisciplinary team of specialists—content experts in their own field, strong communication skills, and interest in teaching and mentoring (14). The COVID-19 ECHO hub team consists of leading subject matter experts: infectious disease specialists, emergency medicine clinicians, the director of Missouri DHSS, an epidemiologist, a rural federally qualified health center director, leadership from the Missouri Hospital Association (MHA), and a pediatric infectious disease clinician. Sharing best practices and ever-evolving information with those caring for the most vulnerable has been necessary to help reduce the spread of the disease and improve patient outcomes. ECHO sessions also proved to be an avenue for sharing concerns and challenges from communities across the state with the DHSS Director, giving him the ability to utilize this information for a more agile state response. Each weekly 60 min conference session consists of a 15–30 min expert didactic presentation, followed by a question and answer session and/or real-life de-identified case-discussions.

Show-Me ECHO utilized internal participant contact database to advertise the project to individuals from the nearly 35 other ECHO topics. They also partnered with the Missouri Primary Case Association, the Missouri Hospital Association, Missouri Nurses Association, Missouri Department of Social Services, and other professional association to attract other participants.

### Collaboration With Public Health Community

On 24 March 2020, the leadership of the Boone County Medical Society (BCMS) initiated a weekly COVID-19 webinar series, together with the medical director of the local Boone County Public Health and Human Services (PHHS) Department. These webinars feature a wide range of topics, and some of the content is repurposed from COVID-19 ECHO, as the rapid need for guidance increased [8]. The BCMS webinars are complementary to the COVID-19 ECHO sessions, especially during “breaking news” events such as the surge in local cases. Many of the speakers from the COVID-19 ECHO are also invited to present their didactics to the BCMS COVID-19 webinars, further expanding the reach of information during this pandemic.

In this paper, we describe the results the pilot project in which we analyzed participants' pre-session information needs survey and questions asked during COVID-19 ECHO. These questions and concerns help us better understand immediate community member information needs during first months of the public health emergency. We also analyzed satisfaction surveys to learn more about the participants' perceived value of the project.

## Context

We conducted a qualitative analysis of two sources of data: (1) responses to a question asked at the time participants registered for COVID-19 ECHO sessions, “What do you anticipate the needs of your community to be? This will help us plan our educational offering”; and (2) transcripts of questions asked by participants during COVID-19 ECHO sessions from March 23 thru May 4, 2020.

For the survey question in an open text format, co-author (MWG) coded responses provided through March 30, 2020. QDAMiner was used to develop codes (15). The first round of coding resulted in 26 codes in four categories. Coding was then shared with another co-author (KGH), who identified coding instances that required additional discussion. MWG and KGH discussed differences in opinion on the codes, resulting in 15 codes in five thematic categories. All disagreements were resolved. MGW and KGH also discussed how codes could be combined to make them more meaningful and developed a revised coding scheme. The remaining responses (registrations March 31- May 4, 2020) were organized by MWG into Excel files and coded using the coding scheme developed in the pilot round. Coding was then shared with KGH, who identified codes that required additional discussion. MWG and KGH discussed differences in opinion on the codes. All disagreements were resolved.

Next, content analysis was completed on the transcripts of questions asked during sessions. We analyzed questions that were captured using the Zoom chat functions during the COVID-19 ECHO sessions. Using the codes developed during the analysis of participants' answers to the survey question, MWG analyzed the participant generated Zoom questions. Although we anticipated new codes and themes to emerge from these data, no new codes were identified. KGH reviewed all codes and identified areas of disagreement. MWG and KGH met to adjudicate differences. Through ongoing discussions, the authors further clarified codes and resulted in complete agreement.

Satisfaction surveys were collected in December 2020, during the MTN bi-annual ECHO survey collection using REDCap (16).

## Details And Results

### Attendance Data

Seven COVID-19 ECHO sessions were held during the study period. An average of 281 participants attended each session (range 202–324). Seven hundred sixty one (*n* = 761) of 1,701 registrants attended at least one of the seven sessions with about half (*n* = 358) non-prescribing clinicians, one fifth non-clinicians (*n* = 159), one fifth physicians (*n* = 153) and remaining participants non-physician prescribers (*n* = 91) (Table 1). Only 60 physicians (39.2 percent) reported their specialty: 15 family and community medicine, 12 pediatrics, 6 internal medicine, 4 emergency medicine, 4 dermatology, and 19 other (nephrology, sleep medicine, obstetrics and gynecology, developmental behavioral pediatrics, etc.) Participants were from 99 out of 114 Missouri counties. The 44 percent COVID-19 ECHO attendance rate is higher than the reported average virtual seminar/webinar attendance rate of 40 percent (17).

**Table 1:** COVID-19 ECHO participant type.

**Category**	**Participant title**	***N***
Non-prescribing clinician (*n* = 358)	Nurse	282
	CHW/Health aide	24
	All oral health professionals	21
	All pharmacy professionals	19
	Other health profession (including therapists)	7
	Mental/behavioral health professional	5
Non-clinician (*n* = 159)	Administrator	89
	Other/Unknown	18
	Other mental/behavioral health professional (including CPS)	16
	Social worker	13
	Student (mostly health professions)	10
	Care coordination	9
	Educator	4
Physician (*n =* 153)	Physician	153
Non-physician prescriber (*n =* 91)	Nurse practitioner	90
	PA	1
		**761**

Didactic presentations were prepared by the hub team experts, who ensured that the topics addressed questions and comments received from participants through pre-surveys or during previous sessions. For example, personal protective equipment was discussed during 4/6/2020 session, critical resources on 4/20/2020 and pediatric presentations of COVID-19 on 5/11/2020 when COVID-19 incidence was peaking, and participants' questions focused mainly on these topics. The DHSS Director held two back-to-back sessions on 6/15/2020 and 6/22/2020 with a brief Missouri state COVID-19 specific updates followed by a question-and-answer session that used most of the hour.

### Thematic Categories

For the seven sessions, 692 (41%) of the registrants responded to the survey question: “What do you anticipate the needs of your community to be?” Many identified more than one concern, which resulted in 1,086 identified needs. Across the seven COVID-19 ECHO sessions, participants asked 315 questions, which also frequently identified more than one concern resulting in 797 identified needs. Concerns fell into five major themes: (1) patient care, (2) information seeking, (3) minimizing exposure, (4) financial themes, and (5) general comments (Table 2).

**Table 2:** Thematic categories.

**Category**	**Identified need**	**Community needs**	**% of comments (*n =* 1,086)**	**Learner questions**	**% of comments (*n =* 797)**
Patient care	Education	137	13%	96	12%
	Symptoms, triage, testing, screen	117	11%	85	11%
	Local access to care and supplies	84	8%	66	8%
	Special populations/care locations	75	7%	54	7%
	Mental wellbeing re covid	66	6%	46	6%
	Other: coordination, home and hospice care, dental	53	5%	44	6%
	*Patient care total*	*532*	*49%*	*391*	*49%*
Minimizing exposure	Prevention, minimizing exposure	52	5%	33	4%
	Telemedicine	29	3%	20	3%
	Worker safety	73	7%	56	7%
	*Minimizing exposure total*	*154*	*14%*	*109*	*14%*
Information	Treatment guidelines	121	11%	108	14%
	Current & accurate	87	8%	66	8%
	Planning for return	45	4%	16	2%
	*Information total*	*253*	*23%*	*190*	*24%*
General comments	General	52	5%	42	5%
	Other	23	2%	18	2%
	*General comments total*	*75*	*7%*	*60*	*8%*
Financial impact	Assistance	47	4%	29	4%
	Burdens	25	2%	18	2%
	*Financial total*	*72*	*7%*	*47*	*6%*
**Grand total**		**1,086**	**100%**	**797**	**100%**

Patient care was the most common concern representing nearly half of survey answers and session questions. Specifically, patient education and identification of symptoms, triage, testing and screening were identified by participants, as the immediate community needs. Other patient care concerns included access to local supplies, mental health and wellbeing as well as care coordination and hospice care. The second most common concern representing nearly one quarter of survey answers and session questions was information seeking. Most of the participants identified treatment guidelines information as an immediate community need, followed by access to current and accurate information and planning for return to work and school. The third most common concern was minimizing exposure. Worker safety was the primary community need identified by the participants, followed by prevention and telemedicine. Participants also voiced concerns about the financial impact of COVID-19 including the burden of lost income and availability of financial assistance. Other, less frequently mentioned concerns related to reflections on individual practices, nursing homes, or schools.

### COVID-19 ECHO Timeline

**Figure 1:**
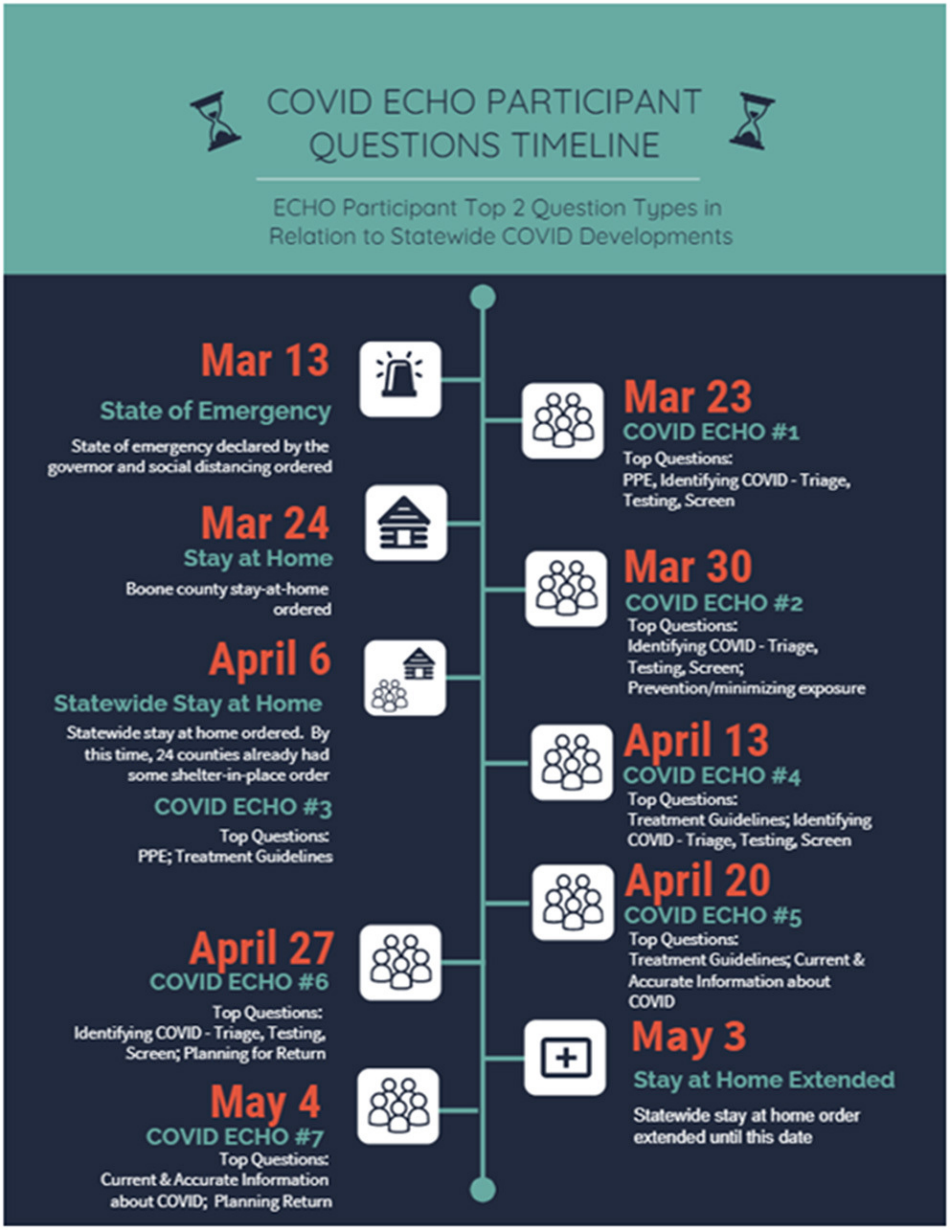
Question timeline.

The first COVID-19 ECHO session was held only 10 days after the State of Emergency declaration by the Governor on March 13, 2020 (Figure 1). Over the 7 weeks, information on patient care and minimizing exposure remained of high interest. We found that most of the questions during the first session concerned the first category—patient care—identification of COVID-19, triage, testing, and screening, as well as personal protective equipment (PPE). The third COVID-19 ECHO session was held immediately after the statewide stay-at-home order. Participants primarily asked questions regarding PPE and treatment guidelines during that session. As the public health situation continued to evolve and change rapidly, participants sought answers mostly from the information category—current and accurate information regarding COVID-19 in session 5.

### Satisfaction Surveys

**Figure 2:**
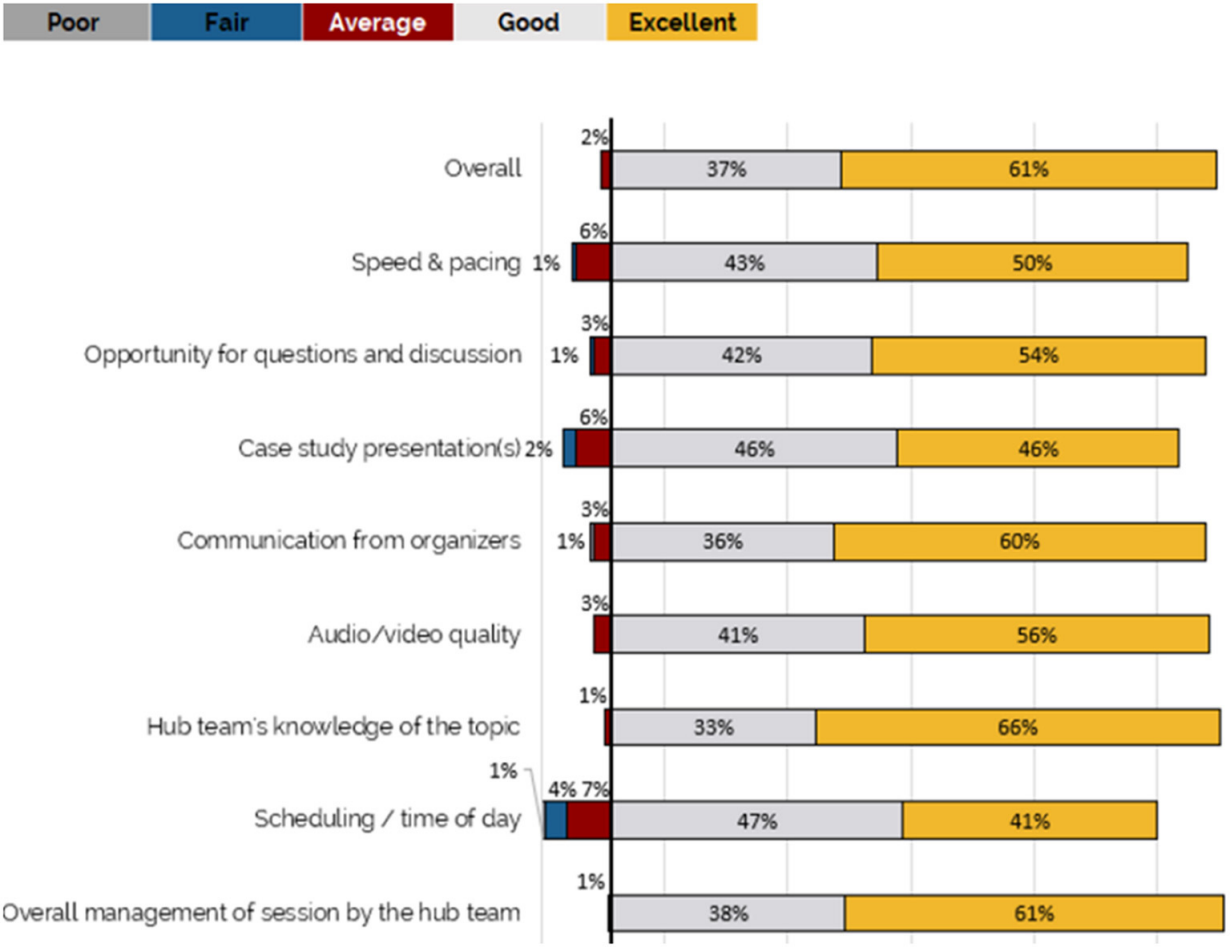
Participant satisfaction.

One hundred seventy attendees (21%) responded to the bi-annual satisfaction survey in December 2020. A five-point Likert scale was used, with possible responses poor, fair, average, good, or excellent. Most attendees rated content quality, as well as logistics and technical operations as good to excellent (Figure 2).

In addition to using the information to improve their own practice, participants also reported sharing the information they learn with peers and community members as well as using information to make changes to policies and protocols across their organization:

“I pass information on to my clients”

“I have been able to share the knowledge I have gained with co-workers and patients. I plan to continue doing so.”

“I share the data that I learn and information ALL the time with parents and staff at my school.”

“I've incorporated the testing recommendation I've learned from this ECHO into my practice.”

“I have used the information to help persuade management to make certain changes in protocol/policy related to COVID-19 and used the information to combat some misinformation around COVID-19 as well.”

“It has been very reassuring to hear the open discussions about COVID-19 in our communities, and to have immediate feedback on what other healthcare providers are seeing and what questions they are grappling with.”

“As an independent school nurse consultant, the COVID-19 series gives me trusted information and experts who are willing to field our questions… this allows me to share trusted information with schools in my area.”

“I felt I received up-to-date information about practices relating to COVID-19. Having access to these experts was so helpful for the rural standpoint […]

## Discussion

The purpose of the COVID 19 ECHO was to provide up to date information to Missourians, and especially rural Missourians. Thus, this small one-site pilot study was intentionally focused on the needs of Missouri communities in the early days of the pandemic. Although very informative for Missouri, this limits generalizability of the results. Sample size of 761 is also small and limits generalizability.

Telehealth technologies play essential role during public health emergencies (12, 18). Some of the known benefits include the ability to provide triage and care to patients without unnecessary exposure, reduce travel burden, provide continuing education, and share information and guidelines in a timely manner (18). Telehealth has also been used to allow quarantined clinicians to continue providing care to their patients remotely (19).

Vulnerable populations, including but not limited to healthcare workers, ethnic and minority groups, rural, immigrant and refugee communities, people with mental illness and chronic diseases, have experienced disproportionate adverse impact of COVID-19 (20). Bhaskar et al. urged a call for action in 2020 to accelerate rapid expansion and adoption of telehealth and telemedicine with the aim of improving access to care, and expanding remote medical education and continuing education (20).

In the environment of misinformation and contradictory reporting, especially in the early days of the pandemic, we found that the COVID-19 ECHO model was responsive to the needs of health care community by sharing up to date knowledge, guidelines, and shared experiences. These sessions were very well-attended, with almost 300 participants from 99 (or 87 percent) of Missouri counties during each session, suggesting its value to health care providers and those caring for vulnerable populations.

COVID-19 recommendations and guidelines change frequently, as more data becomes available almost daily. Clinicians used various virtual platforms to connect and share evidence-based information as well as their personal experiences during the pandemic (21). Providers and others working with vulnerable populations need access to experts to provide up-to-date education and mentoring. Information learned from this pilot project was used to propose and ultimately implement additional ECHO sessions addressing specific population needs: Telemedicine ECHO, MO Moms and Babies, COVID-19 & Kids, Nursing Home COVID-19 Action Network ECHO, and Managing Hospitals & Patients in the Pandemic. To date, these sessions were attended by 1,026 unique participants.

This pilot can help inform strategies for a future pandemic by emphasizing importance of integration of telehealth technologies within current healthcare systems that can be rapidly adjusted to the needs of the community. In addition, understanding categories of immediate learning needs (such as patient care recommendations, ways to minimize exposure, financial burden in patient care, and current and accurate information) can help state officials prepare coordinated and targeted response.

## Data Availability Statement

The raw data supporting the conclusions of this article will be made available by the authors, without undue reservation.

## Ethics Statement

Ethical review and approval was not required for the study on human participants in accordance with the local legislation and institutional requirements. Written informed consent for participation was not required for this study in accordance with the national legislation and the institutional requirements.

## Author Contributions

MB, KE, RM, KH, JM, and AH: study conception and design. EW: data collection. MB, KH, MW-G, EW, C-RS, and RT: data analysis and interpretation of results. All authors: draft manuscript preparation, editing, revising, and approval of final version.

## Funding

MB and C-RS were funded by the National Science Foundation under Grant Number IIS-2027891.

## Conflict of Interest

The authors declare that the research was conducted in the absence of any commercial or financial relationships that could be construed as a potential conflict of interest.

## Publisher's Note

All claims expressed in this article are solely those of the authors and do not necessarily represent those of their affiliated organizations, or those of the publisher, the editors and the reviewers. Any product that may be evaluated in this article, or claim that may be made by its manufacturer, is not guaranteed or endorsed by the publisher.
